# Percutaneous transaxillary approach through the first segment of the axillary artery for the Impella-supported PCI Versus TAVR

**DOI:** 10.1038/s41598-024-51552-3

**Published:** 2024-01-10

**Authors:** Jerzy Sacha, Krzysztof Krawczyk, Witold Gwóźdź, Przemysław Lipski, Wojciech Milejski, Piotr Feusette, Marek Cisowski, Marek Gierlotka

**Affiliations:** 1 Department of Cardiology, University Hospital, Institute of Medical Sciences, University of Opole, Opole, Poland; 2 Faculty of Physical Education and Physiotherapy, Opole University of Technology, Opole, Poland; 3 Department of Cardiac Surgery, University Hospital, Institute of Medical Sciences, University of Opole, Opole, Poland

**Keywords:** Interventional cardiology, Cardiac device therapy

## Abstract

Percutaneous transaxillary approach (PTAX) through the first segment of the axillary artery is not widely recognized as a safe method. Furthermore, PTAX has never been directly compared between Impella-supported percutaneous coronary interventions (Impella-PCI) and transcatheter aortic valve replacement (TAVR). This study evaluated the feasibility and safety of PTAX through the first axillary segment in Impella-PCI versus TAVR. In cases where standard imaging guidance was insufficient, a technique involving puncturing the axillary artery “on-the-balloon” was employed. The endpoints were bleeding and vascular complications, as defined by BARC and VARC-3 criteria. PTAX was successfully performed in all 46 attempted cases: 23 for Impella-PCI and 23 for TAVR. Strict adherence to BARC and VARC-3 criteria led to the frequent identification of major bleeding (57%) and a moderately frequent diagnosis of vascular complications (17%). These incidences were primarily based on post-procedural hemoglobin reduction (> 3 g/dl) but not overt bleeding. The Impella group exhibited a higher rate of BARC 3b bleeding due to a greater hemoglobin decline resulting from the prolonged implant duration and PCI itself. Left axillary access was linked to smaller blood loss. Bleeding and vascular complications, as per BARC and VARC-3 definitions, did not affect short-term prognosis, with only 3 Impella patients succumbing to heart failure unrelated to the procedures during one-month follow-up period.

## Introduction

The percutaneous transaxillary approach (PTAX) constitutes an alternative for large-bore access in patients without femoral access. In this technique, experts from the Society for Cardiovascular Angiography and Interventions (SCAI) and most operators advocate puncturing the second segment of the axillary artery located behind the pectoralis minor muscle, due to its potentially reduced risk of complications^1^. Indeed, there is limited data concerning the safety of the approach through the first axillary artery segment, situated between the lateral border of the first rib and the pectoralis minor muscle^1,2^.

The majority of the data demonstrating the efficiency of PTAX originates from transcatheter aortic valve replacement (TAVR), with a smaller portion coming from procedures involving mechanical circulatory support devices^2–7^. In fact, PTAX outcomes have never been directly compared between Impella-supported percutaneous coronary interventions (PCI) and TAVR. Consequently, our understanding of specific difficulties or complications associated with PTAX, particularly through the first axillary artery segment, in these two clinical contexts remains limited. The undertaking of such a direct comparison also presents challenges, given that PTAX for PCI with Impella and TAVR are typically conducted by distinct groups of operators, specializing either in high-risk PCI or structural heart diseases.

In this study, we analyzed single-center data on PTAX during Impella-supported PCI versus TAVR, both performed through the first segment of the axillary artery by the same team of operators. Our objective was to identify differences in complications related to PTAX between these two clinical scenarios. Additionally, we outline a technique for puncturing the axillary artery “on the balloon” when standard imaging methods do not offer adequate guidance.

## Methods

### Material

The study group consisted of consecutive patients without femoral access who underwent high-risk PCI with Impella support or TAVR using PTAX. The vast majority of patients (96%) had a thorough examination for large-bore arterial access using CT scans, except for those who received the Impella pump for urgent indications, where the access site was chosen based on angiography. All patients presented with significant ilio-femoral arterial disease, which precluded femoral access. Additionally, nine had aortic aneurysms with intraluminal thrombus, and two patients had severely tortuous aortas. In each case, the decision about the treatment approach, particularly regarding the need for mechanical support and the access site, was made by the Heart Team. The procedures were performed by the same group of operators who dealt with both high-risk coronary interventions and structural cardiac procedures. Bleeding and vascular complications were assessed according to BARC and VARC-3 definitions^8,9^. The decrease in hemoglobin level within 3 days after the procedure was considered as bleeding associated with the intervention if no other probable cause existed. All patients provided their written informed consent for the procedures. The study was conducted in compliance with the principles of the Declaration of Helsinki, and the Institutional Ethics Committee of the University of Opole reviewed and approved the study.

### Technique of the percutaneous transaxillary approach

**Figure 1:**
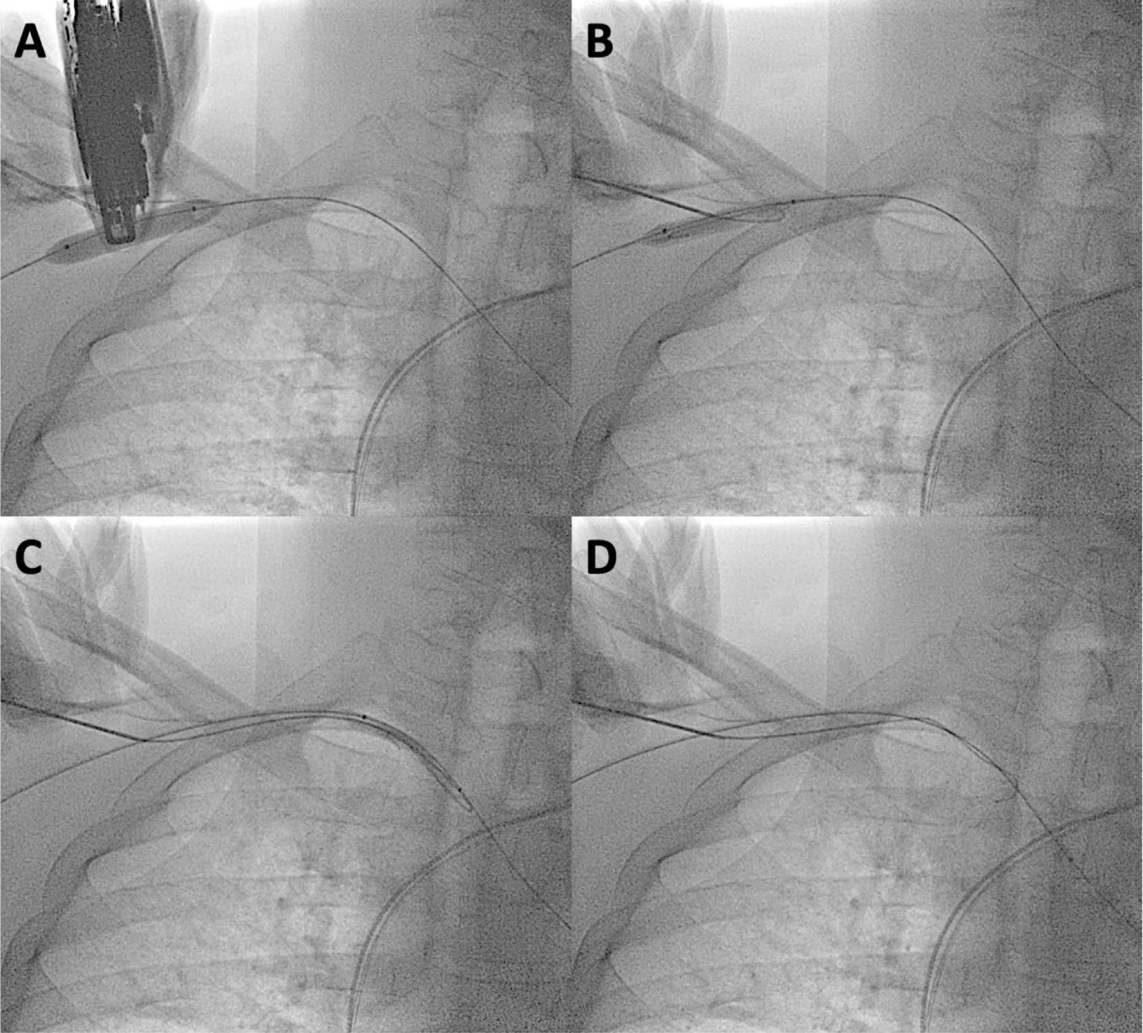
The puncture technique "On the Balloon". (**A**) The peripheral balloon is inserted through radial access and positioned at the puncture site. Subsequently, the artery is punctured together with the balloon under ultrasound guidance. (**B**) The guidewire is inserted into the balloon. (**C**) The guidewire and balloon are jointly advanced to the aorta. (**D**) The guidewire is removed from the balloon and advanced back to the aorta (afterward, the balloon is removed). Refer to supplementary Video S1.

To gain access to the axillary artery, first, a 7F or 6F sheath was placed into the ipsilateral radial artery, and a long 0.035" guidewire was inserted into the ascending aorta, serving as a safety guidewire. Angiography was performed by retrograde contrast injection (diluted with saline 1:1) through the radial sheath to identify the first segment of the axillary artery, i.e., between the clavicle and the thoraco-acromial artery. The arterial puncture was done under ultrasound guidance at a shallow angle to facilitate the insertion of a large sheath. In cases where ultrasound imaging did not provide sufficient visualization, particularly in obese patients, the puncture was performed "on the balloon," as shown in Fig. 1 and the supplementary Video S1. This technique facilitates puncture, as the balloon enhances both ultrasound and fluoroscopic visibility of the puncture site. Moreover, the balloon also enables puncturing a dissected artery, as inflating the balloon compresses the dissected layers, allowing for the needle insertion into the true arterial lumen.

After gaining access, two Proglide/Prostyle sutures were deployed for later percutaneous closure, and then a large Impella or TAVR sheath was inserted. Upon completion of the procedure, a peripheral balloon was introduced through the radial sheath to the access site, with the goal of ensuring arterial tamponade during Impella or large sheath removal. The balloon not only prevented bleeding but also averted arterial stenosis while tightening the Proglide/Prostyle sutures. In case of vascular closure device failure, bleeding was resolved with Angioseal deployment or manual compression along with the balloon tamponade. Alternatively, instead of manual compression, a piece of hemostatic sponge was delivered directly to the bleeding site with the help of Proglide pusher^10^. This double compression, i.e., from inside with the balloon and from outside manually (or with the pusher), for at least 10 min, usually resolved bleeding issues. If all measures failed, the vascular problem was addressed with a stent-graft or self-expanding stent implantation through the radial access^11^.

### Endpoints

The primary endpoints were the differences between the Impella and TAVR groups in bleeding according to BARC type 3 bleeding criteria and major vascular complications according to VARC-3 definition. The secondary endpoints included the differences in minor vascular complications according to VARC-3 definition, as well as various types of BARC and VARC bleeding.

### Statistical analysis

Categorical variables are presented as numeric values and percentages, while continuous variables are given as median and interquartile range (IQR) or mean and standard deviation. The normality of the data distribution was assessed using Shapiro–Wilk test. Differences between variables were tested with either Student's t-test, Mann–Whitney test, or Fisher exact test as appropriate. To identify independent determinants of the primary endpoints, a stepwise multivariable logistic regression was employed, adjusted for age, sex, and BMI. Only procedural variables associated with the primary endpoints in the univariate analysis (with *p* < 0.1) were considered for inclusion in the multivariable models. Similarly, predictors of hemoglobin decline after procedures were determined using multivariable linear regression. The threshold probability of *p* < 0.05 was taken as the level of statistical significance. All analyses were performed using the Statistical Package for Social Sciences (SPSS, v. 22.0, Chicago, IL, USA).

## Results

The study population consisted of 46 patients who underwent PTAX between April 2020 and August 2023. Among them, 23 received the Impella CP (Impella group), while the remaining 23 patients underwent TAVR (TAVR group). Among the TAVR group, 15 subjects received Evolut R or Pro valves, and 8 subjects received Sapien 3 Ultra valves. All attempted PTAX procedures through the first axillary artery segment were successful without the need for conversion to any other approach.

Most patients in the Impella group presented with compromised left ventricular function and Impella was used as mechanical circulatory support for high-risk PCI. While awaiting the procedure, two of them developed cardiogenic shock. Baseline patient characteristics showed significant differences between the Impella and TAVR groups (Table 1). The Impella patients were younger and had a significantly lower left ventricular ejection fraction (LVEF). Severe aortic stenosis was the primary issue among all TAVR subjects, with five patients in the Impella group having the same condition. More patients in the Impella group suffered from unstable angina or non-ST-segment elevation myocardial infarction. The prevalence of left main or three-vessel disease and a history of prior myocardial infarction was also higher among Impella patients. The Syntax Score and EuroScore II indicated a significantly higher surgical risk of death in the Impella group compared to the TAVR group. A greater number of subjects receiving Impella were on dual antiplatelet therapy and beta-blockers. Conversely, TAVR patients were more commonly treated with oral hypoglycemic drugs and antibiotics (for prophylaxis).

**Table 1:** Baseline patient characteristics and pharmacological treatment.

Characteristics	Whole group	Impella group	TAVR group
	(n = 46)	(n = 23)	(n = 23)
Age (years)	74 (68–81)	70 (66–78)*	79 (73–83)*
Male	29 (63)	16 (70)	12 (52)
BMI (kg/m^2^)	26.2 ± 4.5	25.7 ± 4.1	26.7 ± 4.9
Hypertension	42 (91)	19 (83)	23 (100)
Atrial fibrillation	16 (37)	8 (35)	8 (35)
Prior stroke/TIA	9 (20)	5 (22)	4 (17)
Heart failure	46 (100)	23 (100)	23 (100)
NYHA class	2 (2–3)	3 (2–4)	2 (2–3)
proBNP (ng/l)	7217 ± 9122	9257 ± 11,237	4947 ± 5451
Hemoglobin (g/dl)	12.4 ± 1.8	12.6 ± 1.6	12.1 ± 2.0
Creatinine (mg/dl)	1.22 ± 0.91	1.15 ± 0.5	1.3 ± 1.2
LVEF (%)	41 ± 16	32 ± 13‡	50 ± 13‡
Severe AVS	28 (61)	5 (22)§	23 (100)§
Chronic coronary syndrome	7 (15)	5 (22)	2 (9)
Unstable angina	5 (11)	5 (22)*	0*
NSTEMI	7 (15)	7 (30)*	0*
Cardiogenic shock	2 (4)	2 (9)	0
Left main disease	19 (41)	18 (78)§	1 (4)§
Three vessel disease	20 (43)	17 (74)‡	3 (13)‡
Prior myocardial infarction	21 (46)	15 (65)*	6 (26)*
Prior PCI	24 (52)	11 (48)	13 (57)
Prior CABG	5 (11)	4 (17)	1 (4)
LIMA graft	5 (11)	4 (17)	1 (4)
RIMA graft	1 (2)	1 (4)	0
Dyslipidemia	43 (93)	21 (91)	22 (96)
Diabetes mellitus	24 (52)	11 (48)	13 (57)
Chronic kidney disease	30 (65)	15 (65)	15 (65)
COPD	7 (15)	1 (4)	6 (26)
Gastro-intestinal disease	12 (26)	8 (35)	4 (17)
Malignancy	9 (20)	7 (30)	2 (9)
Syntax Score	25.8 ± 20.3	44 ± 10.5‡	7.5 ± 6‡
EuroSCORE II	11.86 ± 15.4	17.91 ± 19.74*	5.8 ± 4.3*
STS Score	6.652 ± 8.698	9.133 ± 11.793	4.171 ± 1.667
Mehran Risk Score (%)	26.1 (26.1–57.3)	26.1 (26.1–57.3)	57.3 (26.1–57.3)
Aspirin	42 (91)	23 (100)	19 (83)
DAPT	37 (80)	23 (100)†	14 (61)†
NOAC	15 (33)	8 (35)	7 (30)
ACEI/ARB	37 (80)	20 (87)	17 (74)
B-blocker	38 (83)	22 (96)*	16 (70)*
Mineralocorticoid antagonist	28 (61)	16 (70)	12 (52)
Diuretics	35 (76)	19 (83)	16 (70)
Statins	44 (96)	23 (100)	21 (91)
SLGT-2 inhibitors	10 (22)	7 (30)	3 (13)
Oral hypoglycemic drugs	14 (30)	3 (13)*	11 (48)*
Insulin	9 (20)	5 (22)	4 (17)
Noradrenaline	5 (11)	4 (17)	1 (4)
Dobutamine	2 (4)	1 (4)	1 (4)
Adrenaline	6 (13)	5 (22)	1 (4)
Levosimendan	2 (4)	2 (9)	0
Antibiotics	36 (78)	14 (61)†	22 (96)†

Values are mean ± standard deviation, median (interquartile range) or n (%). *ACEI* indicates angiotensin-converting enzyme inhibitor; *ARB*, angiotensin receptor blocker; *AVS*, aortic valve stenosis; *BMI*, body mass index; *CABG*, coronary-artery by-pass grafting; *COPD*, chronic obstructive pulmonary disease; *DAPT*, dual antiplatelet therapy; *LIMA*, left internal mammary artery; *LVEF*, left ventricular ejection fraction; *NOAC*, novel oral anticoagulant; *NSTEMI*, non-ST-segment elevation myocardial infarction; *NYHA*, New York Heart Association; *PCI*, percutaneous coronary intervention; *proBNP*, pro B-type natriuretic peptide; *RIMA*, right internal mammary artery; *SGLT-2*, sodium-glucose cotransporter-2; *STS*, Society of Thoracic Surgeons; TIA, transient ischemic attack.

*p < 0.05; †*p* < 0.01; ‡*p* < 0.001; §*p* < 0.0001 for differences between Impella and TAVR group.

Table 2 presents procedural variables and outcomes. Specifically, all PCIs in the Impella group were completed without complications, and some TAVR patients also underwent successful PCI; however, this occurred prior to the valve procedure. The vast majority of TAVRs were carried out without problems. Nevertheless, in one case, a second valve had to be deployed due to partial dislodgement of the primary valve into the left ventricle. Balloon aortic valvuloplasty was commonly performed in the TAVR group. Additionally, five Impella patients with severe aortic stenosis underwent valvuloplasty to unload the left ventricle and facilitate Impella implantation. General anesthesia was more frequently used for TAVR patients, while the volume of intra-procedural fluids, heparin dose, contrast volume, radiation dose, and procedure time (i.e., implant duration) were higher in the Impella group. An increase in creatinine level after the procedure, but not the occurrence of acute kidney injury, was higher in Impella patients. They also had a substantially longer hospital stay.

**Table 2:** Procedural variables and outcomes.

Variables	Whole group	Impella group	TAVR group
	(n = 46)	(n = 23)	(n = 23)
PCI	32 (70)	23 (100)§	9 (39)§
PCI of left main	19 (41)	18 (78)§	1 (4)§
Balloon aortic valvuloplasty	21 (46)	5 (22)†	16 (70)†
General anesthesia	16 (37)	0§	16 (70)§
CTA for access assessment	44 (96)	21 (91)	23 (100)
Completely non-femoral approach	31 (67)	15 (65)	16 (70)
Left axillary access	31 (67)	12 (52)	19 (83)
Minimum axillary artery diameter (mm)	6.5 (6–8)	6.5 (5.5–8)	7 (6–8)
Subclavian-axillary artery angle (degree)	88 ± 16	91 ± 19	84 ± 13
Sheath size	14 (14–21)	14 (14–14)	14 (14–21)*
Proglides/Prostyles usage	46 (100)	23 (100)	23 (100)
Angioseal usage	26 (57)	12 (52)	14 (61)
Vascular closure device failure	3 (7)	2 (9)	1 (4)
Covered stent implantation	3 (7)	2 (9)	1 (4)
Self-expanding stent implantation	3 (7)	2 (9)	1 (4)
Vascular surgery	0	0	0
Brachial plexus injury	0	0	0
Pneumothorax	0	0	0
Periprocedural MI	1 (2)	1 (4)	0
Intra-procedural fluid (ml)	1296 ± 557	1538 ± 660†	1028 ± 208†
Heparin dose (U)	10,000 (7500–12,500)	12,500 (10,000–13,500)‡	7500 (5875–10,000)‡
Protamine usage	20 (43)	8 (35)	12 (52)
Contrast volume (ml)	272 ± 113	327 ± 121‡	218 ± 72‡
Radiation dose (mGy)	1793 ± 1512	2659 ± 1709‡	431 ± 90‡
Implant duration (hours)	2.34 (1.74–3.23)	3.17 (2.5–4.3)‡	1.73 (1.44–2.27)‡
Hemoglobin decline (g/dl)	2.9 ± 1.4	3.4 ± 1.5	2.8 ± 1.1
Time for hemoglobin decline (days after procedure)	2 (1–2.5)	2 (1–3)	2 (1–2.25)
Bleeding from axillary access	1 (2)	1 (4)	0
Large hematoma (> 4 cm)	4 (9)	3 (13)	1 (4)
Red blood cell transfusion	16 (37)	11 (48)	5 (22)
Red blood cell units	0 (0–2)	0 (0–2)	0 (0–0.5)
Hemolysis	1 (2)	1 (4)	0
Creatinine increase after procedure (mg/dl)	0.02 ± 0.31	0.12 ± 0.33*	-0.7 ± 0.25*
Acute kidney injury	5 (11)	4 (17)	1 (4)
PM/CRT/ICD implantation	7 (15)	5 (22)	2 (9)
Infection/sepsis	7 (15)	5 (22)	2 (9)
Hospital stay (days)	15 (7–25)	20 (14–26)†	7 (6–17)†
One-month MI/stroke/TIA	0	0	0
One-month mortality	3 (7)	3 (13)	0

Values are mean ± standard deviation, median (interquartile range) or n (%), and median (lower–upper limit) for sheath size. *CRT*, indicates cardiac resynchronization therapy; *CTA*, computed tomography angiography; *ICD*, implantable cardioverter-defibrillator; *MI*, myocardial infarction; *PCI*, percutaneous coronary intervention; *PM*, pacemaker; *TIA*, transient ischemic attack.

*p < 0.05; †*p* < 0.01; ‡*p* < 0.001; §*p* < 0.0001 for differences between Impella and TAVR group.

There was no need for vascular surgery, and neither group experienced stroke, brachial plexus injury, or pneumothorax. In the entire study population, only three stent-grafts and three self-expanding stents were implanted, as a result of vascular closure device failure and arterial dissection. Three deaths unrelated to PTAX occurred in the Impella group: one due to cardiogenic shock and two from heart failure (one of them after discharge).

**Figure 2:**
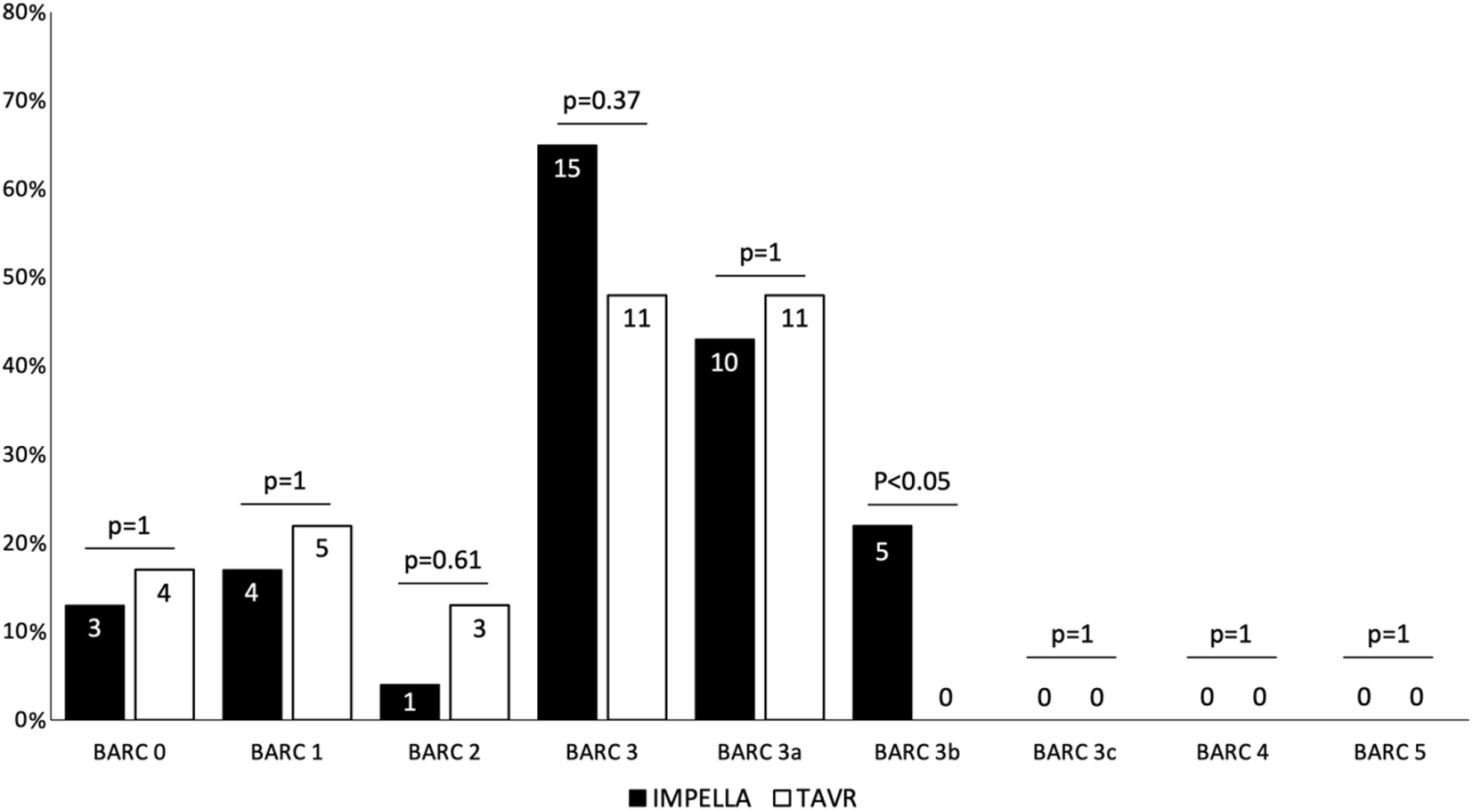
The prevalence of different bleeding types according to the Bleeding Academic Research Consortium (BARC) in patients undergoing Impella implantation and transcatheter aortic valve replacement (TAVR).

**Figure 3:**
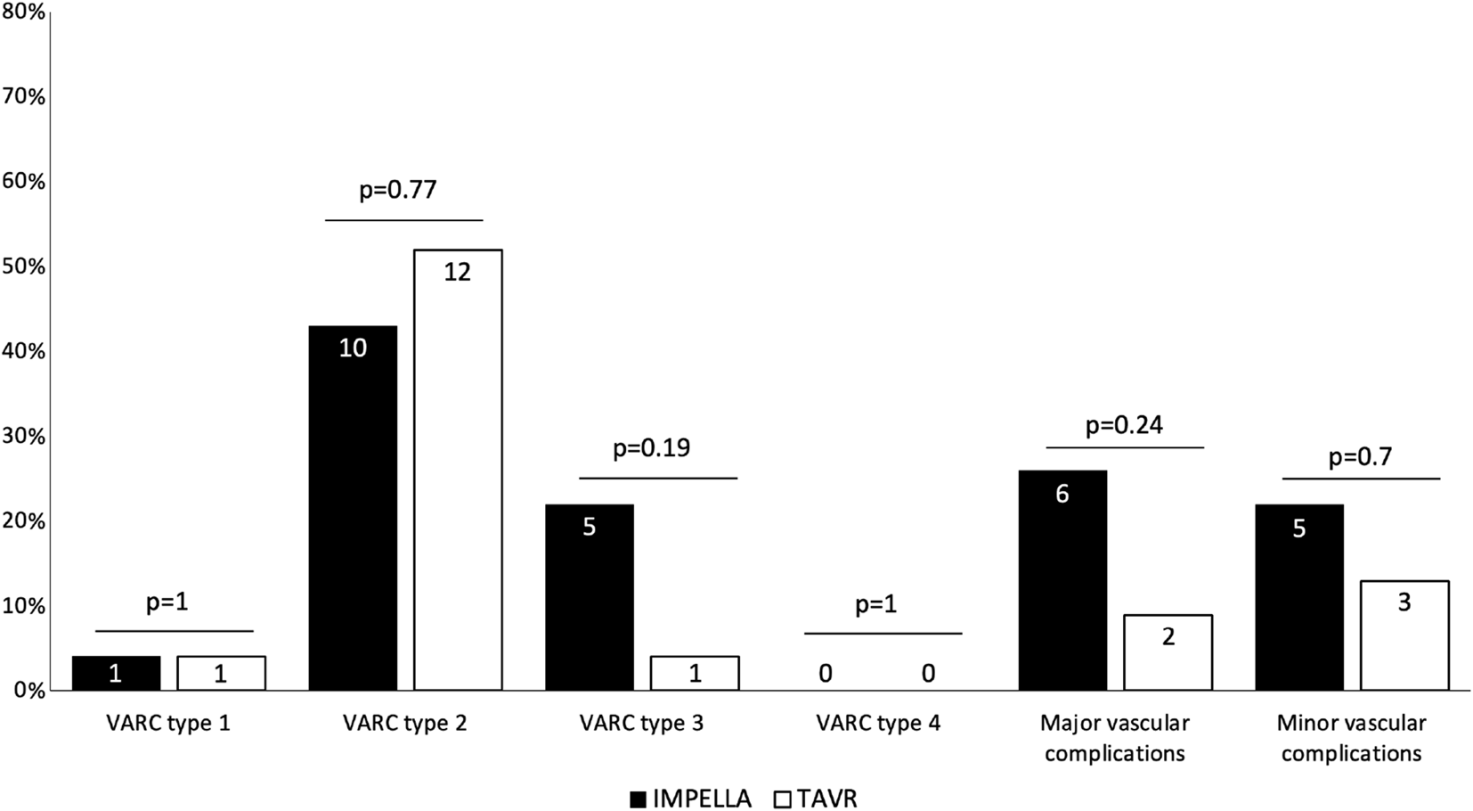
The prevalence of various types of bleeding (type 1–4), as well as major and minor vascular complications according to the Valve Academic Research Consortium 3 (VARC) in patients undergoing Impella implantation and transcatheter aortic valve replacement (TAVR).

Figures 2 and 3 present the prevalence of bleeding and vascular complications according to BARC and VARC-3 definitions. Various types of bleeding were primarily identified based on the drop in hemoglobin level but not on the occurrence of overt bleeding. The median time for the greatest hemoglobin decline was 2 days (IQR: 1–2.5 days) after the procedure. Primary endpoints, namely BARC 3 bleeding and major vascular complications (according to VARC-3 criteria), were observed in the entire study population at rates of 57% and 17%, respectively. There was no significant difference between the Impella and TAVR groups in these outcomes. However, the Impella patients experienced a significantly higher incidence of BARC 3b bleeding. Table 3 displays the independent determinants of BARC 3 bleeding, as well as the determinants of hemoglobin decline following the procedure. Since the number of major vascular complications was less than 10 (specifically, 8), conducting a reliable multivariable analysis for this outcome was not feasible.

**Table 3:** Multivariable analysis for hemoglobin decline and BARC type 3 bleeding.

Hemoglobin decline			
Variables	Multivariable linear regression		
	B	95% CI	*P* value
Implant time (hours)	0.041	0.014–0.067	0.003
Left axillary access	− 1.036	− 1.809 to − 0.264	0.01
BARC type 3			

Variables	Multivariable logistic regression		
	OR	95% CI	*P* value
HB decline after procedure (g/dl)	5.39	1.85–15.7	0.002
RBC transfusion (units)	3.13	1.15–8.55	0.026
Age (years)	0.93	0.9–0.98	0.002

The candidates for the multivariable models were selected based on the univariate analysis of all procedural variables from Table 2, where: (i) implant duration, bleeding from axillary access, left axillary access and balloon aortic valvuloplasty (BAV) were associated with hemoglobin (HB) decline; (ii) BAV, hemoglobin decline, red blood cell (RBC) transfusion, and left axillary access were linked to BARC type 3 bleeding. Each model was adjusted for age, sex, and BMI.

## Discussion

In this study, PTAX was utilized in patients undergoing the Impella CP implantation or TAVR. All procedures were successfully conducted through the first segment of the axillary artery by the operators who performed both Impella-supported PCI and valve interventions. Bleeding complications, as per BARC 3 criteria, were frequent and led to the recognition of major vascular complications in some patients, as defined by VARC-3. No discernible difference was observed between the two groups in this respect. Due to inherent reasons, patients requiring mechanical circulatory support with the Impella pump needed to maintain the axillary access for a longer duration, resulting in a larger hemoglobin decline and consequently, a significantly higher incidence of BARC 3b bleeding. However, the blood loss did not lead to a worse prognosis, and there were no procedure-related deaths. Over the one-month observation period, three patients in the Impella group died due to heart failure.

Several aspects of this study warrant further consideration. Specifically, the first segment of the axillary artery proved to be a secure access site for large bore devices. This approach was feasible in each case, with no need for conversion to alternative access sites. No patient required vascular surgery, nor did anyone experience brachial plexus injury or pneumothorax. Moreover, contrary to other reports that used the first axillary segment for endovascular aortic procedures, none of our patients suffered a stroke^12,13^. However, attributing such a difference to the specifics of aortic versus cardiac interventions is challenging, and it should rather be considered a matter of chance. Proglides/Prostyles were employed for access site closure in all patients. Additionally, Angioseals were used, mainly to stop oozing, in 26 subjects (57%). Three patients (7%) needed covered stent implantation due to the failure of vascular closure devices. This is a relatively low number compared to other reports where, for example, covered stents were used in up to 44% of cases, and indeed, they constituted an essential aspect of PTAX^5^. However, our data shows that covered stenting should only remain a bailout procedure, to be employed when balloon and manual compression, or compression with a hemostatic sponge and Proglide pusher, cannot effectively address the bleeding issue^10^.

In 46% of the study population, hemoglobin loss ranged from 3.0 to 5.0 g/dl (BARC 3a). Furthermore, in 11% of the whole group, blood loss exceeded 5 g/dl (BARC 3b). However, overt bleeding from the axillary site occurred in only one case and was successfully managed with prolonged manual compression. In the multivariable analysis, the decrease in hemoglobin level was directly associated with the implant duration; conversely, the use of the left axillary access was linked to a smaller blood loss. The diagnosis of BARC 3 bleeding was independently determined by the hemoglobin decline and red blood cell (RBC) transfusion, and it was inversely related to patient age. The implant time in Impella patients was significantly longer than in TAVR subjects, and the hemoglobin decline was partially attributed to the PCI itself. Moreover, the volume of intra-procedural fluid infusion was larger in the Impella group, which might lead to some blood dilution. Additionally, more patients with Impella received dual antiplatelet therapy, and the heparin dose was also larger in this group. Therefore, the higher incidence of BARC 3b bleeding probably did not solely result from the Impella insertion through the percutaneous transaxillary access but was rather influenced by the aforementioned factors. This is supported by the fact that neither hemoglobin drop nor BARC 3 bleeding were independently associated with Impella usage itself. It should also be noted that two patients in the Impella group developed cardiogenic shock while awaiting Impella-supported PCI. As the analysis was conducted on an intention-to-treat basis, these patients were not excluded from the study. However, it is important to consider that patients with cardiogenic shock typically experience different complications and outcomes. According to our study, the left axillary access, instead of the right access, seems to be a safer way to avoid blood loss during PTAX.

Compared to other reports, complications were more frequent in our material. In a recent systematic review, major bleeding and major vascular complications were reported at rates of 2.7% and 2.8%, respectively, while blood transfusion and stent-graft implantation occurred in 5.5% and 10.9%, respectively. The discrepancy between the complication rates and the therapeutic interventions in this review was attributed to the underreporting of clinical outcomes, heterogeneous outcome definitions, and a lack of adjudication ^2^. According to the VARC-2 and VARC-3 definitions, major vascular complications should be recognized when vascular events lead to major bleeding, i.e., when the hemoglobin decline associated with the procedure exceeds 3 g/dl^9,14^. Indeed, instances of bleeding and vascular complications seem to be underreported. In a study where 44.1% of patients received stent-grafts, major vascular complications were noted in 14.3% of cases^5^. In a propensity-matched analysis of percutaneous versus surgical transaxillary access in TAVR, major vascular complications occurred in 3.0% versus 1.5% of the respective groups; however, the exact definition for these complications is not provided in the report^4^. In another study where the average hospital stay was 1.2 days, with 83% of cases being discharged the following day, no bleeding or vascular complications were documented^6^. In our analysis, the median time for the highest hemoglobin decline was 2 days (IQR: 1–2.5 days) after the intervention. Therefore, accurate detection of the hemoglobin drop during a one-day hospitalization is not feasible. In a recent multicenter registry addressing PTAX for Impella pumps, a low rate of vascular complications was reported; however, the authors used the first edition of VARC definition, which did not include hemoglobin loss exceeding 3 g/dl as a criterion for major vascular complications^7,15^.

In our study, we strictly adhered to the BARC and VARC-3 criteria, and a significant portion of bleeding complications were identified solely based on laboratory examinations leading to the recognition of major vascular complications in some subjects. Yet, a high prevalence of these complications in our patients did not impact their prognosis, challenging the usefulness of these criteria in subjects undergoing PTAX. The practicality of BARC and VARC scores in patients treated with mechanical circulatory support (i.e., Impella pumps) was questioned in a recent review. The authors highlighted a series of major limitations of these scores, particularly in the context of the intensive care unit^16^. Indeed, the BARC and either VARC-2 or VARC-3 criteria are not limited to overt bleeding; they also include hematomas and procedural manipulations, such as the insertion and removal of large sheaths, which can lead to a decline in hemoglobin. However, as our study indicates, the high rate of recognizing these outcomes doesn't necessarily correlate with patient prognosis.

Regarding other types of complications, it is worth mentioning the low prevalence of acute kidney injury, i.e., 13% in the entire group. The incidence of kidney injury was particularly low in the TAVR group (5%), despite a very high risk according to the Mehran risk score (median 57.3%; IQR 26.1–57.3). The renal protection offered by PTAX during TAVR was highlighted in a recent meta-analysis, where this approach reduced the risk of kidney damage by 43% compared to intrathoracic approaches (i.e., transaortic or transapical)^17^.

So far, PTAX was mainly compared with the surgical axillary approach^2^. To the best of our knowledge, this is the first report directly comparing PTAX between two different invasive procedures, namely Impella implantation and TAVR. Furthermore, these two types of interventions were carried out through the first axillary artery segment by the same team of operators, allowing us to specifically assess whether the access complications differed between these two distinct clinical scenarios. According to our data, the only notable difference was the higher prevalence of BARC 3b bleeding in the Impella group; however, its cause is likely multifactorial, and it did not impact the prognosis. Despite the favorable outcomes presented in this manuscript, transaxillary access should not be overused, primarily due to the previously reported increased risk of stroke associated with this procedure in TAVR patients^18,19^.

## Limitations

The single-center retrospective design and short-term outcomes limit the broader generalizability of our findings. Further studies are needed to validate the safety of PTAX as an alternative large-bore access in patients without femoral access.

## Conclusion

PTAX through the first segment of the axillary artery for Impella-supported PCI, when compared to TAVR, does not differ in terms of BARC 3 bleeding and major vascular complications. However, interventions with Impella present a higher incidence of BARC 3b bleeding, likely linked to PCI and the duration of axillary access maintenance. Left axillary access, in comparison to the right side, is associated with a lower risk of blood loss. In terms of short-term prognosis, PTAX through the first axillary artery segment yields favorable outcomes.

### Supplementary Information

Supplementary Video 1.

## Data Availability

The data that support the findings of this study are available from the corresponding author upon reasonable request.
